# Biochemical Profile of *Limnospira platensis* Cultivated Under Elevated CO_2_ with the Addition of Sodium Selenite

**DOI:** 10.3390/plants15182804

**Published:** 2026-09-13

**Authors:** Elizaveta M. Kovalenko, Marina E. Vavilkina, Anatoly V. Grigorenko, Maksim A. Kravets, Mikhail S. Vlaskin

**Affiliations:** Joint Institute for High Temperatures, Russian Academy of Sciences, 125412 Moscow, Russia; vavilkina-marina@rambler.ru (M.E.V.); presley1@mail.ru (A.V.G.); kravets.ma@phystech.edu (M.A.K.)

**Keywords:** selenium biofortification, biomass productivity, biochemical composition, elemental profile, microalgae

## Abstract

Selenium (Se) biofortification of microalgae is a promising strategy for producing functional biomass with enhanced nutritional value. This study evaluates the effect of sodium selenite (Na_2_SeO_3_) supplementation to the culture medium on the biochemical profile of *Limnospira platensis* cultivated under intensive growth conditions with an elevated CO_2_ concentration (3 vol.%). Two consecutive cultivation experiments were conducted using Na_2_SeO_3_ supplementations of 10, 20, 40, and 80 mg/L along with a control (0 mg/L). In the second experiment, biomass previously cultivated at 20 mg/L served as the inoculum to evaluate the response of a pre-adapted culture. Biomass productivity, protein, carbohydrate, lipid, pigment, CHNS composition, Se accumulation, and macro- and microelement contents were determined. Maximum biomass productivity (0.369 g·L^−1^·day^−1^) was obtained at 10 mg/L. The most severe disruptions to biochemical and elemental parameters occur at 80 mg/L. This concentration triggers a statistically significant reduction in protein content (down to 46.98% in the first experiment), an accumulation of mercury, an elevation in carbohydrates, and a corresponding shift in the C/N ratio. The second experiment demonstrated enhanced metabolic stability of the pre-adapted culture, including preservation of protein content under high selenium stress and normalization of copper accumulation, indicating the adaptive capacity of *L. platensis* to prolonged selenium exposure. The concentration of Se in the biomass grown under 0 (control), 10, 20 and 80 mg/L of Na_2_SeO_3_ was 0.5, 88.7, 394.0 and 762.0 mg/kg, respectively. Overall, a method for producing *L. platensis* biomass with a controlled Se content has been demonstrated. Based on these findings, Na_2_SeO_3_ concentrations of 10–40 mg/L are recommended for producing Se-enriched biomass, balancing productivity, nutritional quality, and elemental safety. The nutritional efficacy of the biomass obtained using this method should be the subject of future research.

## 1. Introduction

*Limnospira platensis* (Gomont) (commercially known as “spirulina”) is a multicellular filamentous cyanobacterium cultivated worldwide in open raceway ponds and closed photobioreactors [1]. Its natural habitat comprises tropical and subtropical alkaline lakes with pH 8–11 and high carbonate/bicarbonate concentrations [2,3]. For commercial purposes, *L. platensis* is cultivated in both open raceway ponds and closed photobioreactors of various designs.

*L. platensis* biomass is widely used in food, feed, pharmaceuticals, and cosmetology due to its high nutritional value and diverse biological effects [4,5]. The FAO considers it a promising protein source for global food security, and it is utilized as a dietary supplement for anemia, immune disorders, and dyslipidemia [6,7].

The biomass contains 60–70% protein with a complete amino acid profile, 5–10% lipids (including γ-linolenic acid), 15–25% carbohydrates (including immunostimulatory sulfated polysaccharides) [7], and valuable pigments such as C-phycocyanin (5–15%), chlorophyll *a*, and carotenoids [8]. Phenolic compounds range from 2 to 20 mg GAE/g and correlate with antioxidant activity [9,10].

*L. platensis* exhibits high plasticity in mineral metabolism, actively accumulating macro- and microelements (K, Na, Ca, Mg, Fe, Zn, Cu, Mn, I) from the medium [11,12]. This property makes it a convenient platform for targeted biofortification with essential microelements—particularly selenium—by introducing inorganic salts into the culture medium, enabling natural biotransformation into more bioavailable organic forms [13,14].

Selenium (Se, atomic number 34) is an essential microelement for mammals, being incorporated into 25 selenoproteins identified in humans [15]. These include glutathione peroxidases (GPx1–GPx4), thioredoxin reductases (TrxR1–3), and iodothyronine deiodinases, which accounts for the key role of selenium in cellular antioxidant defense, thyroid function regulation, and immune response maintenance [16,17]. The daily allowance for selenium is 50–55 μg for adults (WHO) [18]; in diagnosed deficiency states, therapeutic doses of 100–400 μg/day are applied. Selenium deficiency affects, by various estimates, 15–40% of the global population and is particularly characteristic of regions with selenium-poor soils, such as Central and Northern Europe, China, and New Zealand [19,20].

The multifaceted significance of selenium for human health has been confirmed by an extensive evidence base of clinical and epidemiological studies. Large randomized controlled trials demonstrated a reduction in the risk of developing colorectal, lung, and prostate cancers upon supplementation with inorganic selenium [21]. However, the SELECT study (Selenium and Vitamin E Cancer Prevention Trial) revealed no significant protective effect of the organic form against prostate cancer, indicating a form-dependent mechanism of action for this microelement [22]. The prospective KiSel-10 study demonstrated a decrease in oxidative stress markers and an improvement in cardiovascular indices during the co-administration of Se and coenzyme Q10 [23]. The bioavailability and biological activity of organic selenium forms (Se-methionine, Se-cysteine, Se-methylselenocysteine) exceed those of inorganic forms (sodium selenite, sodium selenate), which establishes the priority of developing organic sources of the microelement [24]. Se deficiency is associated with neurodegenerative changes and cognitive impairment, which substantiates its neuroprotective role [14].

*L. platensis* can transform inorganic Na_2_SeO_3_ into organic Se forms (primarily Se-methionine and Se-cysteine) by incorporating it into proteins, lipids, and polysaccharides [25]. Se-enriched spirulina exhibits a range of documented pharmacological effects. It was demonstrated in [26] hat Se-containing phycocyanin (Se-PC) isolated from Se-enriched *L. platensis* enhances photodynamic therapy for lung cancer, increasing tumor inhibition to 90.4% compared to 53.1% achieved with conventional phycocyanin [27]. Furthermore, Se-enriched spirulina combined with antitumor drugs (docetaxel, oxaliplatin, topotecan) enhanced apoptosis and reduced proliferation in colorectal and prostate cancer cells in vitro, and suppressed tumor growth in a xenograft model in vivo [28]. Comparative studies in rats showed that Se-enriched spirulina restores tissue Se concentrations during dietary deficiency more effectively than inorganic selenite, whereas ${\mathrm{Na}}_{2}{\mathrm{SeO}}_{3}$ provided a higher recovery of antioxidant defense enzyme activities (GPx, TrxR) [29,30]. The inclusion of Se spirulina in the diet of juvenile Asian seabass (Lates calcarifer) increased GPx activity and immunological indices, while concurrently reducing mortality under infectious challenge [31]. It has been shown that when ${\mathrm{Na}}_{2}{\mathrm{SeO}}_{3}$ is introduced into Zarrouk’s medium at concentrations of 0.5–40 mg/L, *Limnospira platensis* maintains normal growth and efficiently transforms inorganic selenium into organic forms, predominantly selenomethionine (SeMet) and selenocysteine (SeCys). At a concentration of 40 mg/L, the selenium content in the biomass can reach ~1200 μg/g of dry weight, with protein and chlorophyll levels remaining substantially unchanged [25]. In [32] Se spirulina exhibited dose- and time-dependent Se accumulation (organic forms, proteins + polysaccharides). While non-toxic alone, it potentiated DOC, OXA, and TOP anticancer activity in colorectal and prostate cancer cells (increased apoptosis, ROS, reduced proliferation) and suppressed tumor growth in vivo.

Biomass enrichment can be performed both within the range of stimulating ${\mathrm{Na}}_{2}{\mathrm{SeO}}_{3}$ concentrations (0.5–40 mg/L), which do not inhibit culture growth and ensure organic Se accumulation up to 400–1200 μg/g of dry weight, and at higher concentrations using stepwise administration schemes [13,25,33]. Optimization of cultivation conditions—including medium composition, light intensity, and light spectrum—allows for an increase in both Se bioaccumulation and the antioxidant activity of the biomass [9,13]. Beyond the biological activity of the Se-enriched biomass, optimizing the conditions for its production is of substantial interest. A key aspect of the industrial production of spirulina is the carbon dioxide nutrition of the culture. *L. platensis* efficiently utilizes ${\mathrm{CO}}_{2}$ during photosynthesis, rendering it a promising candidate for biological ${\mathrm{CO}}_{2}$ mitigation [34,35]. Elevated ${\mathrm{CO}}_{2}$ concentrations (1–5 vol.%) in the gas phase stimulate biomass growth and ensure pH stabilization within the optimal range for spirulina (8.5–9.5) [36,37]. Previously, under conditions of 3 vol.% ${\mathrm{CO}}_{2}$ in closed photobioreactors with continuous illumination, a daily biomass productivity of *L. platensis* of up to $329\;\mathrm{mg}\cdot L^{-1}\cdot {\mathrm{day}}^{-1}$ was achieved [38]. The utilization of flue and waste gases as a source of ${\mathrm{CO}}_{2}$ represents a promising avenue in green biotechnology [35,37], while operation at atmospheric ${\mathrm{CO}}_{2}$ levels has demonstrated the fundamental feasibility of using air as the sole carbon source [39].

Since the accumulation of proteins, pigments, and other biologically active compounds is tightly coupled with the metabolic rate of the culture, it can be hypothesized that cultivating spirulina in closed photobioreactors under elevated CO_2_ levels may enhance the content of biologically active compounds and improve the biochemical characteristics of the biomass, as well as increase the efficiency of its biofortification with microelements, including selenium.

However, data on the combined effect of elevated CO_2_ levels and ${\mathrm{Na}}_{2}{\mathrm{SeO}}_{3}$ supplementation on the biochemical composition of *L. platensis* biomass are lacking in the literature. Existing studies on the biochemical composition of Se-enriched spirulina have been conducted exclusively under atmospheric ${\mathrm{CO}}_{2}$ conditions, and data regarding changes in the pools of proteins, lipids, carbohydrates, pigments, and phenolic compounds under the combination of these two factors have not been reported. In our previous work, the growth kinetics of *L. platensis* and the toxicity of ${\mathrm{Na}}_{2}{\mathrm{SeO}}_{3}$ at concentrations of 20–640 mg/L under 3 vol.% CO_2_ conditions were investigated. The acute toxicity threshold (80–100 mg/L) was established, and the range of 0–20 mg/L was shown to be optimal for maintaining culture viability. The scientific novelty of the present work lies in the study of the biochemical composition of Se-enriched *Limnospira platensis* cultivated under an elevated ${\mathrm{CO}}_{2}$ concentration.

The aim of the present study was to investigate the biochemical composition of *Limnospira platensis* biomass—including proteins, lipids, carbohydrates, the pigment complex (chlorophyll a, carotenoids, phycocyanin), and phenolic compounds, as well as elemental (CHNS) and mineral trace profiles—as a function of sodium selenite concentration under elevated ${\mathrm{CO}}_{2}$ condition (3 vol.%).

## 2. Results

### 2.1. Biomass Yield

The biomass yield per single photobioreactor ($M_{1\mathrm{PBR}}$) and the average daily biomass productivity (V) of spirulina across the two experiments are presented in Table 1. As shown in the table, increasing the concentration of sodium selenite in the cultivation medium led to a progressive reduction in biomass yield and average daily culture productivity. The highest biomass yield was observed at a ${\mathrm{Na}}_{2}{\mathrm{SeO}}_{3}$ concentration of 10 mg/L in the first experiment, whereas further increases in selenium concentration resulted in marked growth inhibition.

For each treatment, biomass from all replicate photobioreactors was pooled, filtered through one sieve, and dried in one tray. M_1_PBR was calculated as total pooled biomass divided by the number of reactors. Due to this pooling procedure, variability measures are not available for yield data. The combined filtration and drying procedure introduced an estimated measurement uncertainty of approximately 5%.

Table 1 also defines the sample designations for the obtained biomass lots, which are used throughout the subsequent text.

### 2.2. Protein Content in Spirulina Biomass

**Figure 1:**
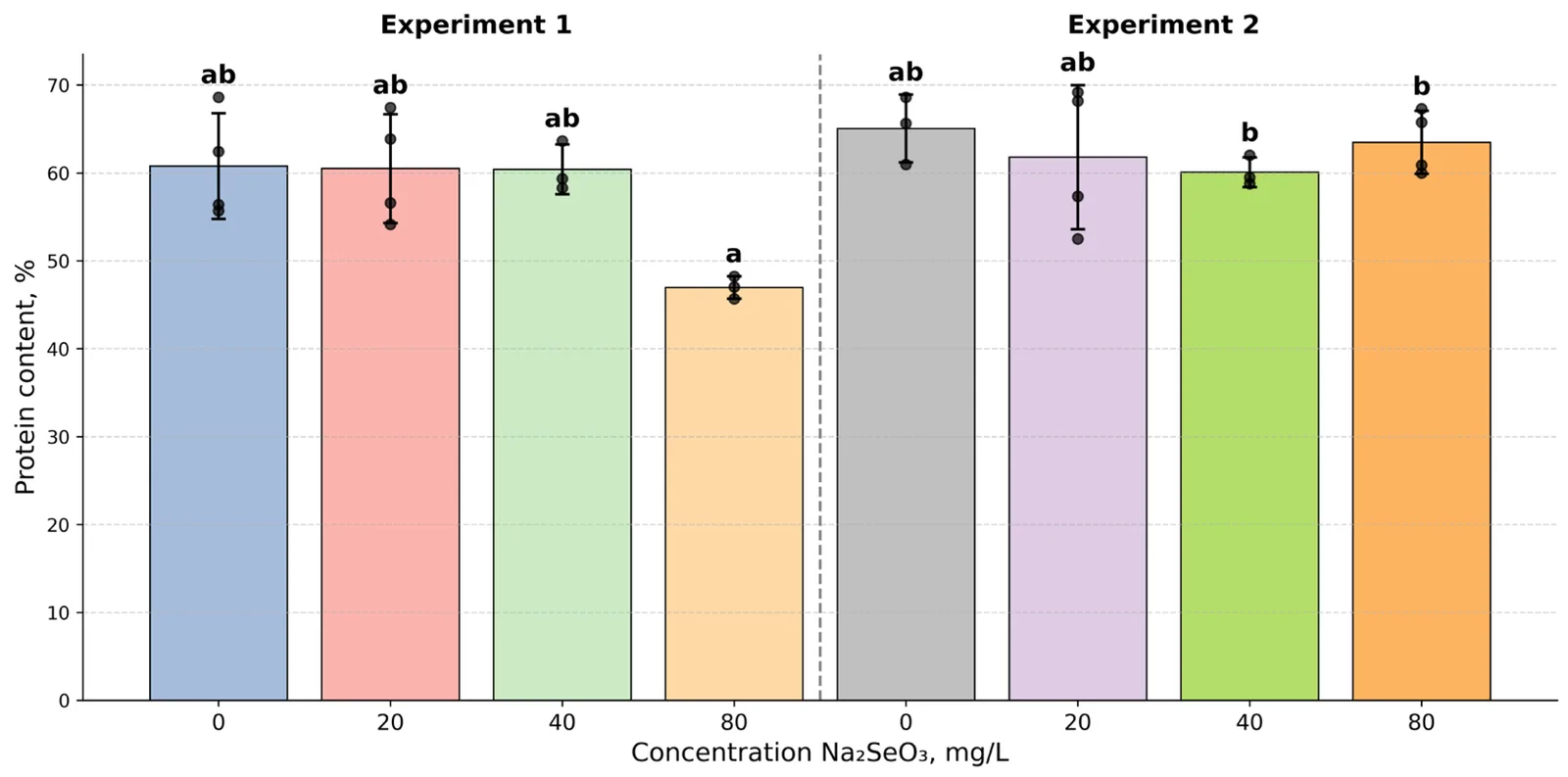
Protein content in Spirulina biomass under different Na_2_SeO_3_ supplementations (mean ± standard deviation, *n* = 3–4). Columns represent mean values, error bars indicate SD, and dots represent individual measurements. Different lowercase letters above the bars denote statistically significant differences (*p* < 0.05).

The protein content in the spirulina biomass for samples subjected to various ${\mathrm{Na}}_{2}{\mathrm{SeO}}_{3}$ supplementations is shown in Figure 1. One-way analysis of variance (ANOVA) revealed statistically significant differences in protein content between the experimental treatments ($F\left(7,19\right)=3.58$; p=0.012). However, Levene’s test indicated a violation of the homogeneity of variances (p=0.006). Therefore, a robust Welch’s ANOVA was performed to validate the findings, which confirmed the presence of significant differences ($F\left(7,8.31\right)=20.26$; p<0.001). Post-hoc pairwise comparisons using the Games–Howell test (with Holm’s correction) revealed that the Se80_1_ sample (46.98±1.27%) had a statistically significantly lower protein content compared to both the Exp2 Se40 treatment (60.09±1.71%, p=0.019) and the Exp2 Se80 treatment (63.49±3.59%, p=0.031). Differences between the remaining treatments did not reach statistical significance (p>0.05). Thus, in the first experiment, the high selenium concentration (80 mg/L) led to a significant reduction in protein content compared to the corresponding concentrations in the second experiment.

### 2.3. Lipid Content in Spirulina Biomass

**Figure 2:**
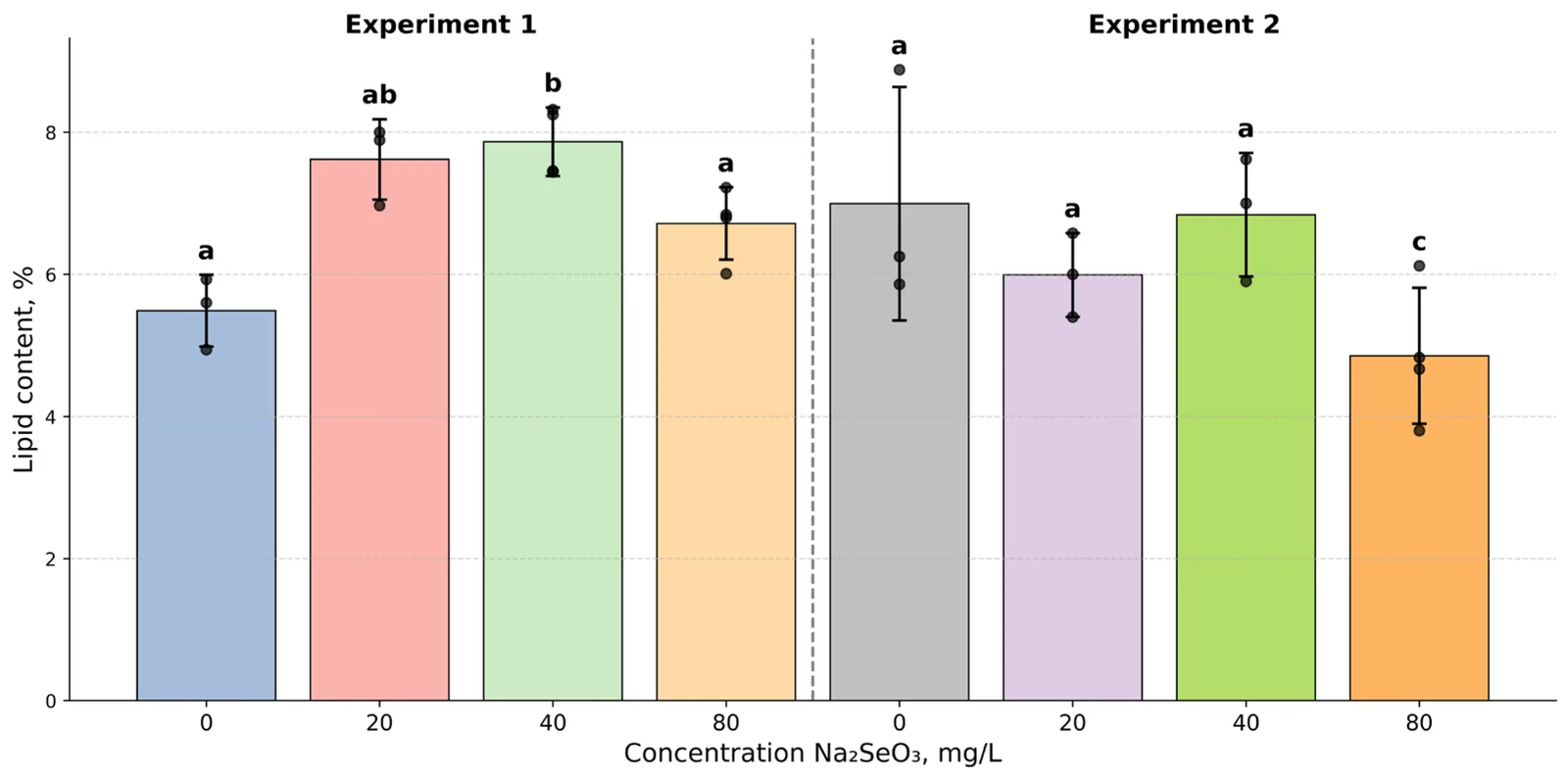
Lipid content in spirulina biomass under different Na_2_SeO_3_ supplementations (mean ± standard deviation, *n* = 3–4). Columns represent mean values, error bars indicate SD, and dots represent individual measurements. Statistical analysis was performed using one-way ANOVA followed by Tukey’s HSD post-hoc test (α = 0.05). Different lowercase letters above the bars denote statistically significant differences: groups sharing a common letter are not significantly different; groups without a common letter differ significantly. All other pairwise comparisons did not reach statistical significance (*p* > 0.05).

The lipid content in the spirulina biomass for samples with various ${\mathrm{Na}}_{2}{\mathrm{SeO}}_{3}$ supplementations is shown in Figure 2. One-way ANOVA revealed statistically significant differences in lipid content among the treatments ($F\left(7,19\right)=5.67$; p=0.0012). Tukey’s post-hoc test demonstrated that in the first experiment, a selenium concentration of 40 mg/L (7.87±0.48%) significantly increased lipid levels compared to the control group (5.49±0.50%; p=0.023). Concentrations of 20 and 80 mg/L showed no significant differences from the control (p>0.05). Conversely, a decrease in lipid content was observed in the second experiment at 80 mg/L (4.86±0.96%) relative to its respective control (7.00±1.64%; p=0.050). Inter-experimental comparisons established that both the 20 mg/L group from the first experiment (7.62±0.57%) and the 40 mg/L group from the first experiment (7.87±0.48%) significantly exceeded the 80 mg/L group from the second experiment (4.86±0.96%), with p=0.006 and p=0.001, respectively. The remaining pairwise comparisons did not reach the significance level (p>0.05). Consequently, the effect of selenium on lipid accumulation depends on the concentration and potentially on the experimental conditions: in the first trial, moderate doses stimulated accumulation, whereas a high dose exerted an inhibitory effect in the second trial.

### 2.4. Carbohydrate Content in Spirulina Biomass

**Figure 3:**
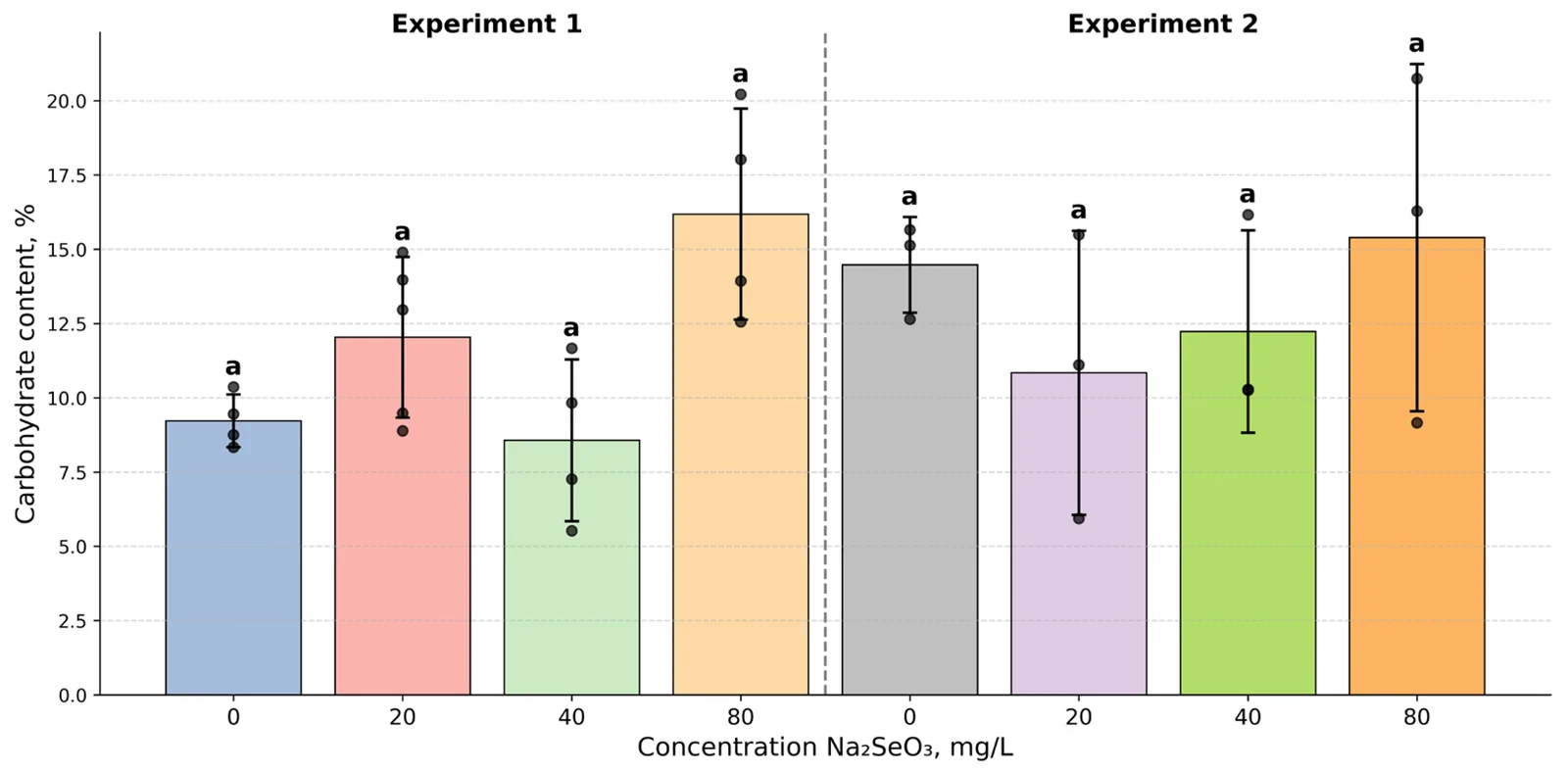
Carbohydrate content in spirulina biomass under different Na_2_SeO_3_ supplementations (mean ± standard deviation, *n* = 3–5). Columns represent mean values, error bars indicate SD, and dots represent individual measurements. Statistical analysis was performed using one-way ANOVA followed by Tukey’s HSD post-hoc test (α = 0.05). Different lowercase letters above the bars denote statistically significant differences: groups sharing a common letter are not significantly different; groups without a common letter differ significantly. All pairwise comparisons not indicated by different letters did not reach statistical significance (*p* > 0.05).

The carbohydrate content in the spirulina biomass cultivated under different sodium selenite concentrations is shown in Figure 3. Although one-way ANOVA suggested a potential effect of sodium selenite concentration on carbohydrate content (*F*(7,23) = 2.61; *p* = 0.042), post-hoc pairwise comparisons did not confirm statistically significant differences between individual treatment groups (Tukey’s HSD, all *p*-adj > 0.05; Kruskal–Wallis test, *p* = 0.084).

### 2.5. Phycocyanin Content in Spirulina Biomass

**Figure 4:**
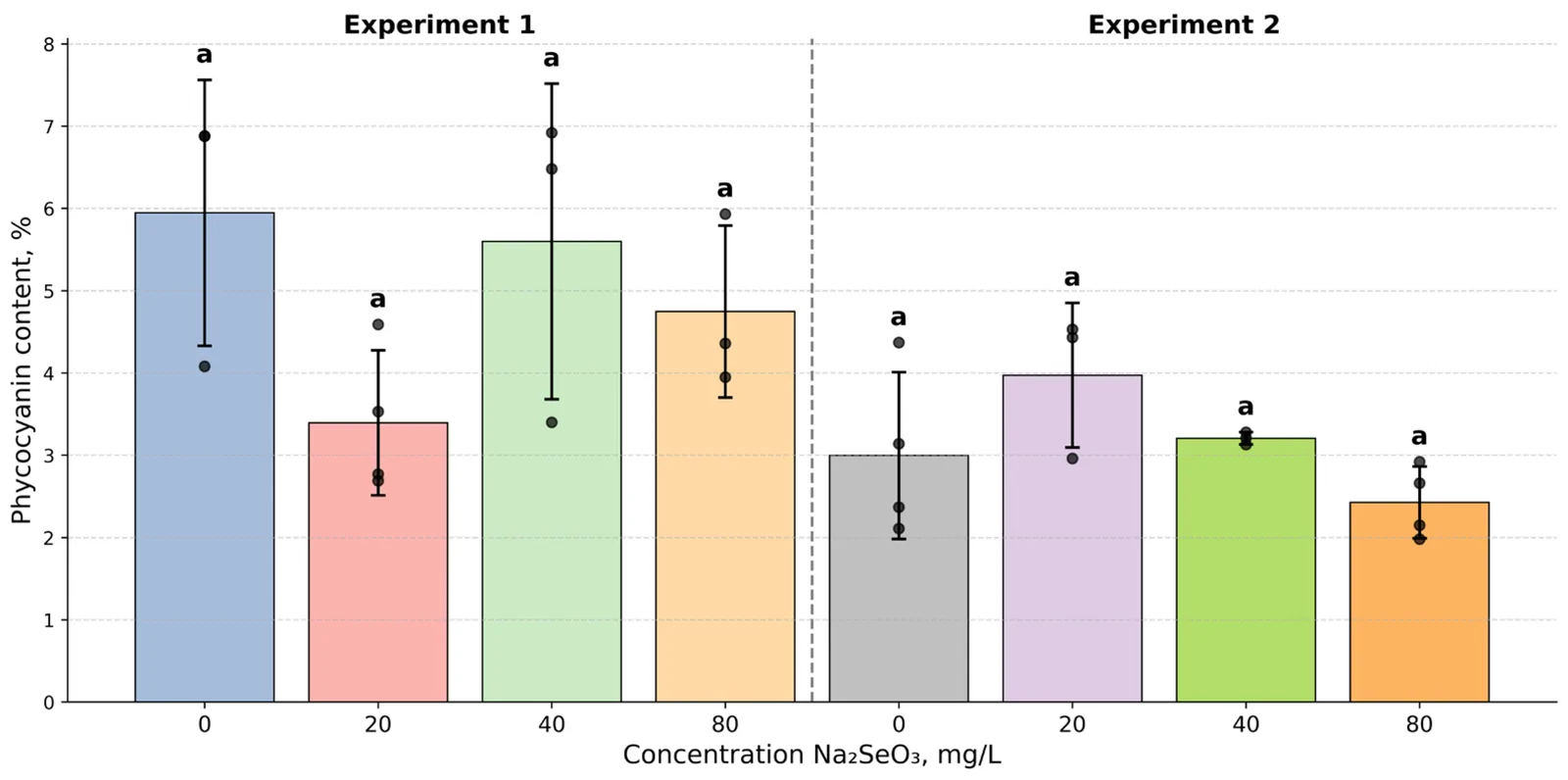
Phycocyanin content in spirulina biomass under different ${\mathrm{Na}}_{2}{\mathrm{SeO}}_{3}$ supplementations (mean ± standard deviation, n=3–4). Columns represent mean values, error bars indicate SD, and dots represent individual measurements. Statistical analysis was performed using the Kruskal–Wallis test followed by pairwise comparisons with Benjamini–Hochberg FDR correction. The same letter a above all bars indicates the absence of significant pairwise differences.

The phycocyanin content in the spirulina biomass cultivated under various sodium selenite concentrations is presented in Figure 4. Prior to statistical analysis, outliers identified by the interquartile range (IQR) method were removed from the dataset, yielding 3–4 replicates per group. Phycocyanin content showed overall differences among groups by Kruskal–Wallis test (H = 15.93, *p* = 0.026); however, Benjamini–Hochberg FDR-corrected pairwise comparisons revealed no significant differences between individual treatments (all *p* > 0.05).

### 2.6. Phenolic Content in Spirulina Biomass

**Figure 5:**
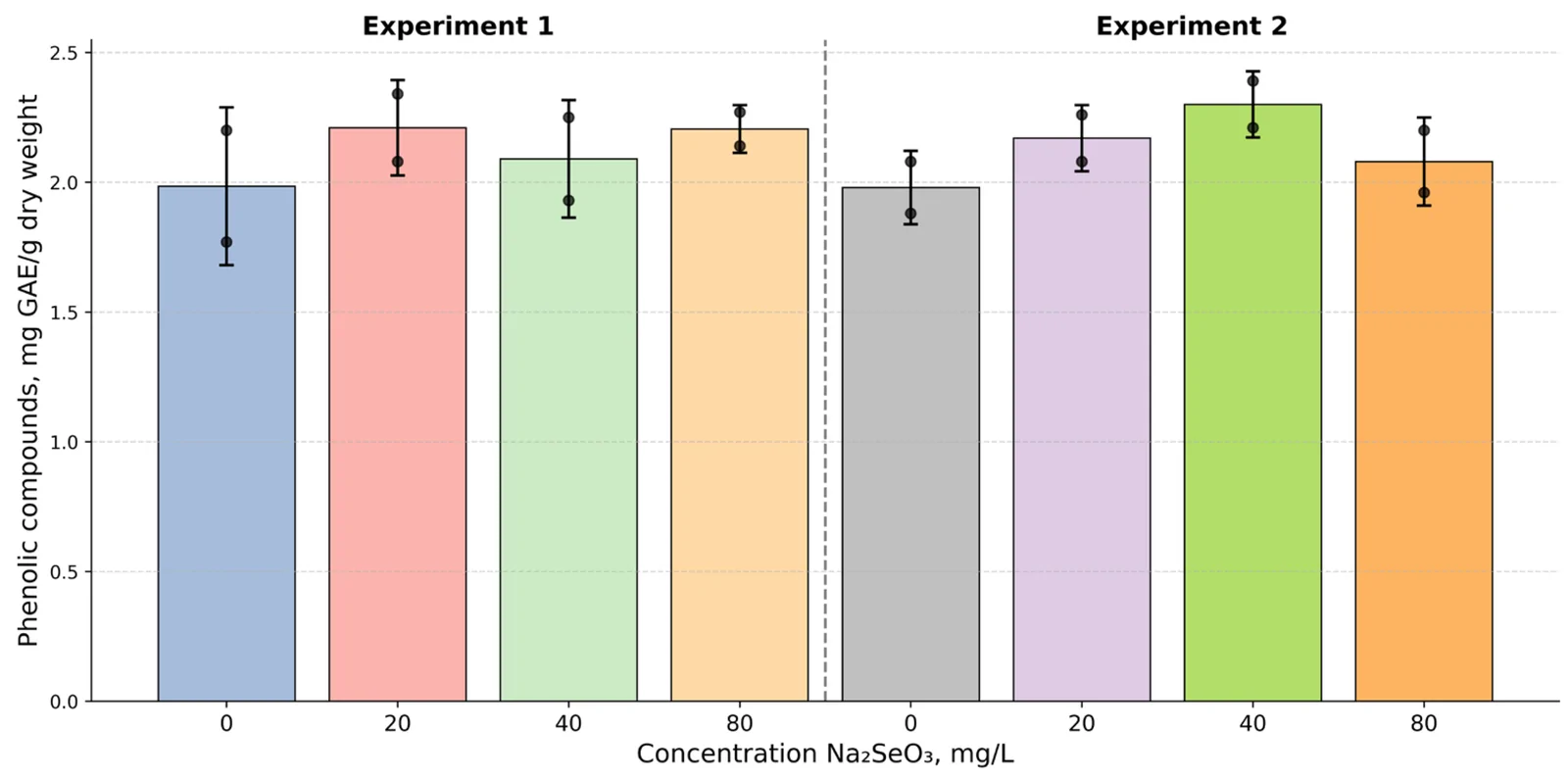
Phenolic content in spirulina biomass under different ${\mathrm{Na}}_{2}{\mathrm{SeO}}_{3}$ supplementations. Error bars correspond to the standard deviation (SD, n=2). Dots represent individual replicate measurements.

Total phenolic content varied within a narrow range (1.77–2.39 mg GAE/g dry weight) across all treatments, with no clear dose-dependent trend (Figure 5).

### 2.7. Trace Element Composition

Table 2 presents the dependence of major and trace element concentrations in the spirulina biomass on ${\mathrm{Na}}_{2}{\mathrm{SeO}}_{3}$ supplementation. The data show that the spirulina biomass is highly enriched in macro- and microelements such as sodium, magnesium, phosphorus, sulfur, potassium, calcium, and iron.

#### 2.7.1. Selenium Content in the Biomass

**Figure 6:**
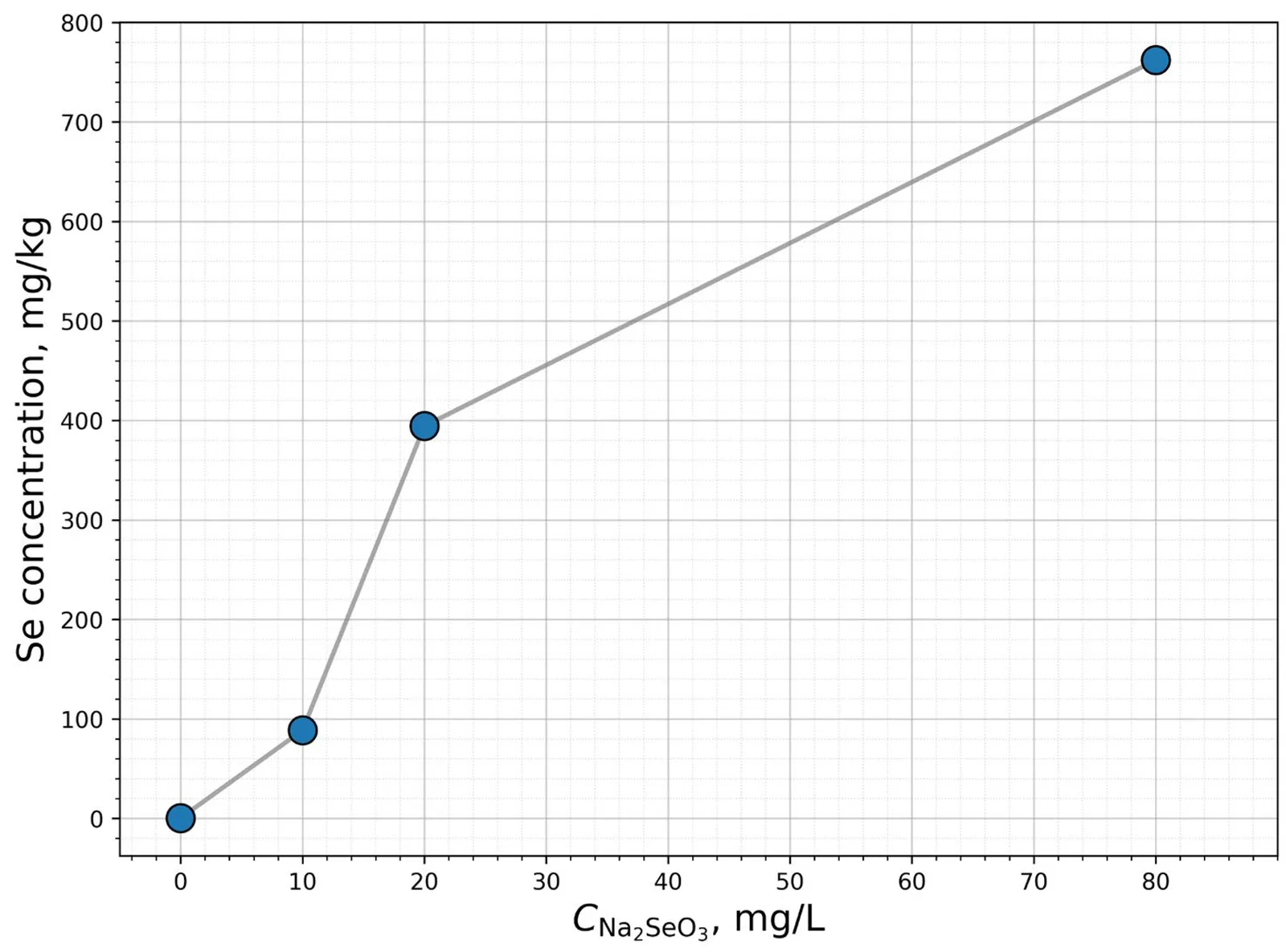
Selenium content in spirulina biomass samples depending on ${\mathrm{Na}}_{2}{\mathrm{SeO}}_{3}$ supplementation to the culture medium (based on data from Experiment 1).

The selenium content in the spirulina biomass for samples with different ${\mathrm{Na}}_{2}{\mathrm{SeO}}_{3}$ additions is shown in Figure 6. The selenium concentration in the biomass of the control sample (Se0_1_) was 0.5 mg/kg of dry weight. With the addition of 10 mg/L ${\mathrm{Na}}_{2}{\mathrm{SeO}}_{3}$ to the cultivation medium, the Se content increased to 88.7 mg/kg; at 20 mg/L, it reached 394.0 mg/kg; and at 80 mg/L, it rose to 762.0 mg/kg. Thus, a clear dose-dependent increase in selenium accumulation is observed with increasing concentrations of the element in the medium.

In the second experiment at 20 mg/L (Exp2), the selenium content in the biomass reached 439.0 mg/kg, which is close to the value obtained in the first experiment at the same concentration (394.0 mg/kg), demonstrating the reproducibility of the results. The slightly higher selenium value in the biomass during the second experiment is also explained by the presence of selenite carried over within the inoculum, whereas the inoculum for the first experiment was completely devoid of sodium selenite.

#### 2.7.2. Heavy Metal Content in the Biomass

**Figure 7:**
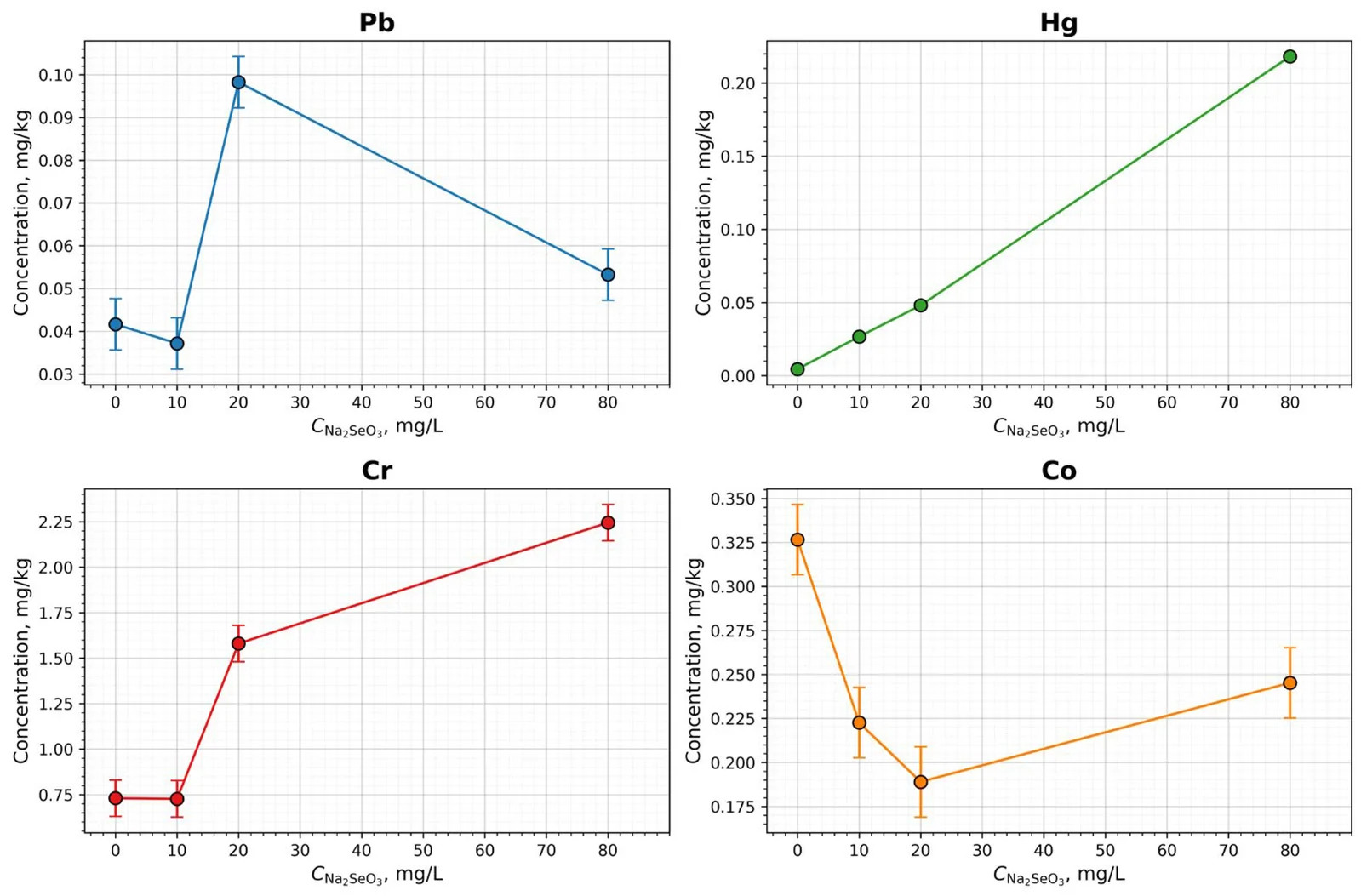
Heavy metal content for samples under various ${\mathrm{Na}}_{2}{\mathrm{SeO}}_{3}$ supplementations. Each bar represents a single sample measurement; error bars indicate analytical method uncertainty.

The concentrations of lead, mercury, chromium, and cobalt in the spirulina biomass are presented in Figure 7. The lead content across all investigated samples did not exceed the maximum allowable level for dietary supplements established by European Commission Regulation (EU) 2023/915 (3.0 mg/kg). Cadmium and nickel concentrations remained well within regulatory limits (1.0 mg/kg for cadmium according to Regulation (EU) 2023/915; for nickel, the calculated daily intake did not exceed the tolerable daily intake (TDI) established by EFSA). Chromium and cobalt levels were also within acceptable safety thresholds for food products and dietary supplements, confirming their non-toxicity. The mercury content in samples cultivated at selenite concentrations of 0, 10, and 20 mg/L did not exceed permissible limits. At 80 mg/L ${\mathrm{Na}}_{2}{\mathrm{SeO}}_{3}$, however, the mercury concentration reached 0.22 mg/kg, which exceeds the maximum allowable level established by the EC Regulation (0.10 mg/kg), though it remains below the advisory limit set by the Codex Alimentarius (1.0 mg/kg).

#### 2.7.3. Macro- and Microelement Content in Spirulina Biomass

**Figure 8:**
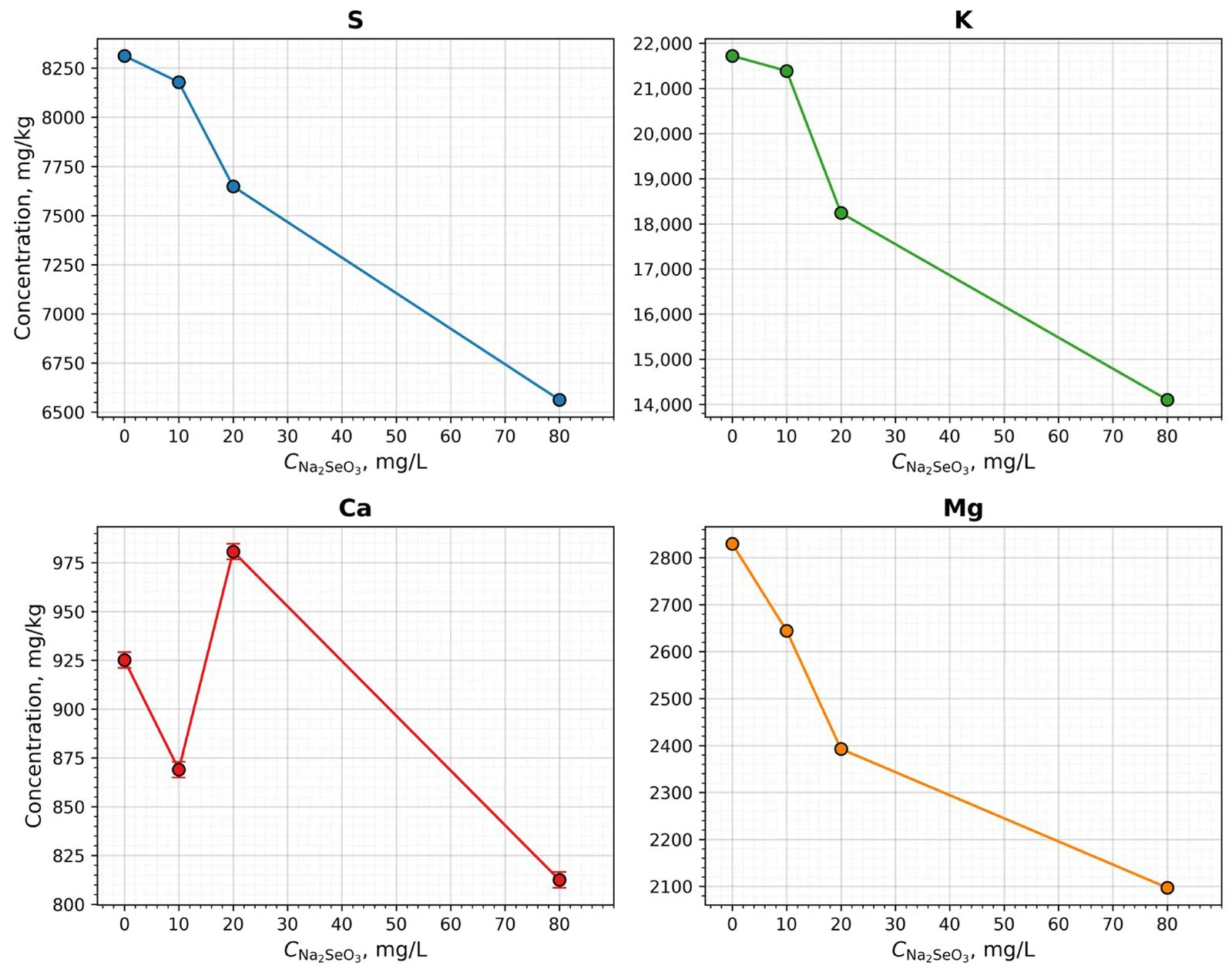
Macroelement content in spirulina biomass cultivated under various Na_2_SeO_3_ concentrations. Each bar represents a single sample measurement; error bars indicate analytical method uncertainty.

The concentrations of macroelements (sulfur, potassium, calcium, and magnesium) in the spirulina biomass are presented in Figure 8. Analysis of the macroelement profile revealed that as the sodium selenite concentration in the medium increased, the most pronounced and consistent reductions occurred in sulfur, potassium, and magnesium. The sulfur content decreased from 8312 μg/g in the control to 6563 μg/g at 80 mg/L; potassium fell from 21,722 to 14,101 μg/g; and magnesium dropped from 2830 to 2097 μg/g. Thus, all three elements exhibited a parallel downward trend, characterized by a substantial decline at high doses of selenite. In the second experiment at 20 mg/L, the values for sulfur (7346 μg/g) and potassium (18,393 μg/g) occupied an intermediate position between the control and the corresponding group from the first experiment. Conversely, magnesium (2696 μg/g) nearly recovered to control levels, which may indicate that this inhibitory effect is less stable at moderate selenite concentrations. The calcium content did not exhibit a clear, dose-dependent trend.

**Figure 9:**
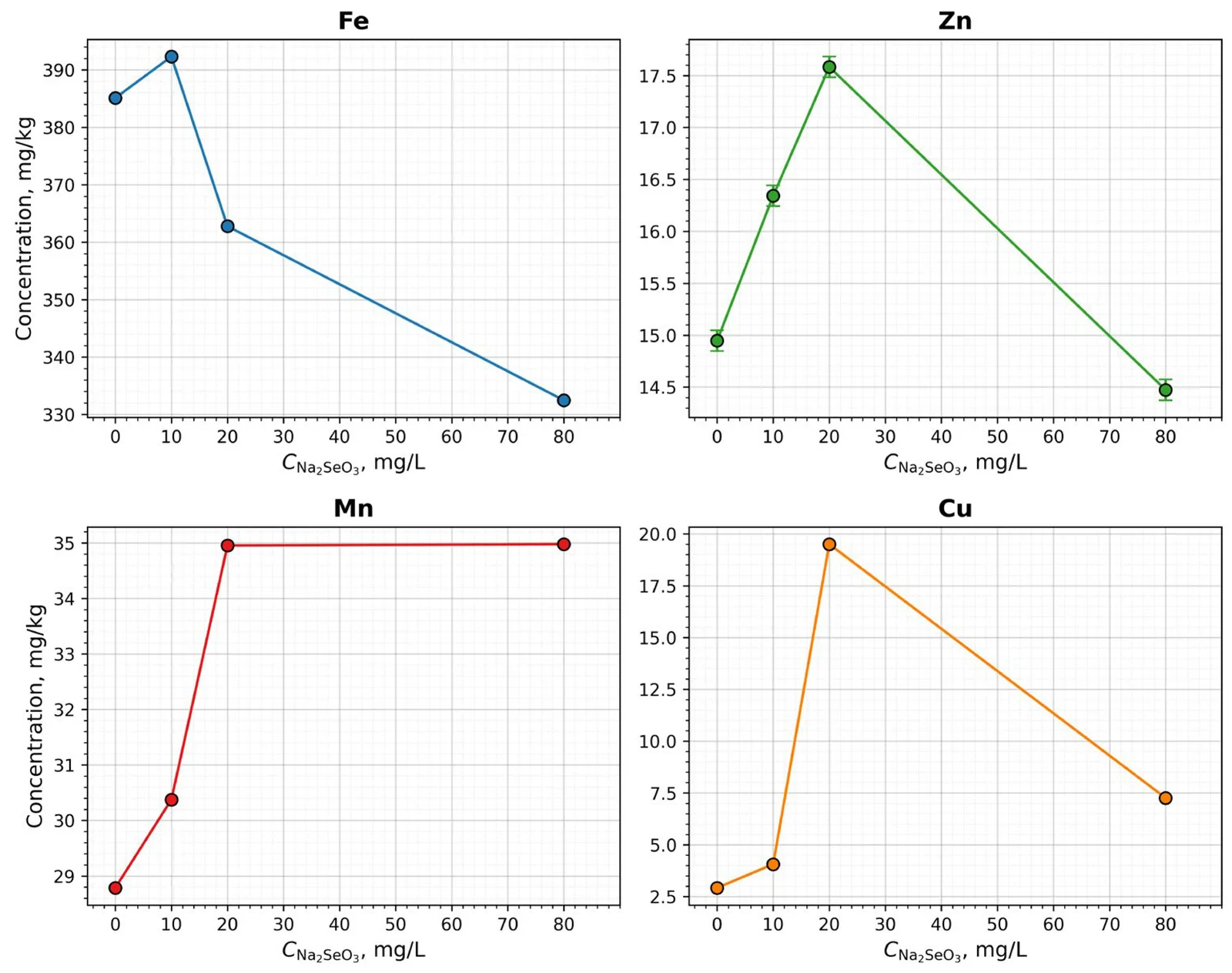
Microelement content in spirulina biomass cultivated under varying Na_2_SeO_3_ concentrations. Each bar represents a single sample measurement; error bars indicate analytical method uncertainty.

Among the microelements (Figure 9), iron showed a moderate decline at sodium selenite concentrations of 20 and 80 mg/L. Manganese and zinc concentrations lacked a clear dose-dependent pattern. Copper displayed an anomalous profile: at 20 mg/L in the first experiment, its concentration rose sharply to 19.5 mg/kg, whereas at 10 and 80 mg/L, it remained within the range of 4–7 mg/kg. In the second experiment—conducted using biomass pre-adapted to 20 mg/L sodium selenite—the copper content decreased to 3.2 mg/kg, closely aligning with control values and those observed at other concentrations. It is possible that the sharp increase in copper during the first experiment was associated with an initial stress response to selenium supplementation, which was subsequently resolved in the continuous cultivation of the adapted culture. The recorded values for Fe, Mn, Zn, and Cu (excluding the anomalous data point) fall within the ranges described in the literature for spirulina. Overall, the results of the second experiment demonstrate that the selenium-adapted biomass maintains an elemental profile close to that of the control samples.

### 2.8. Results of CHNS Analysis

**Figure 10:**
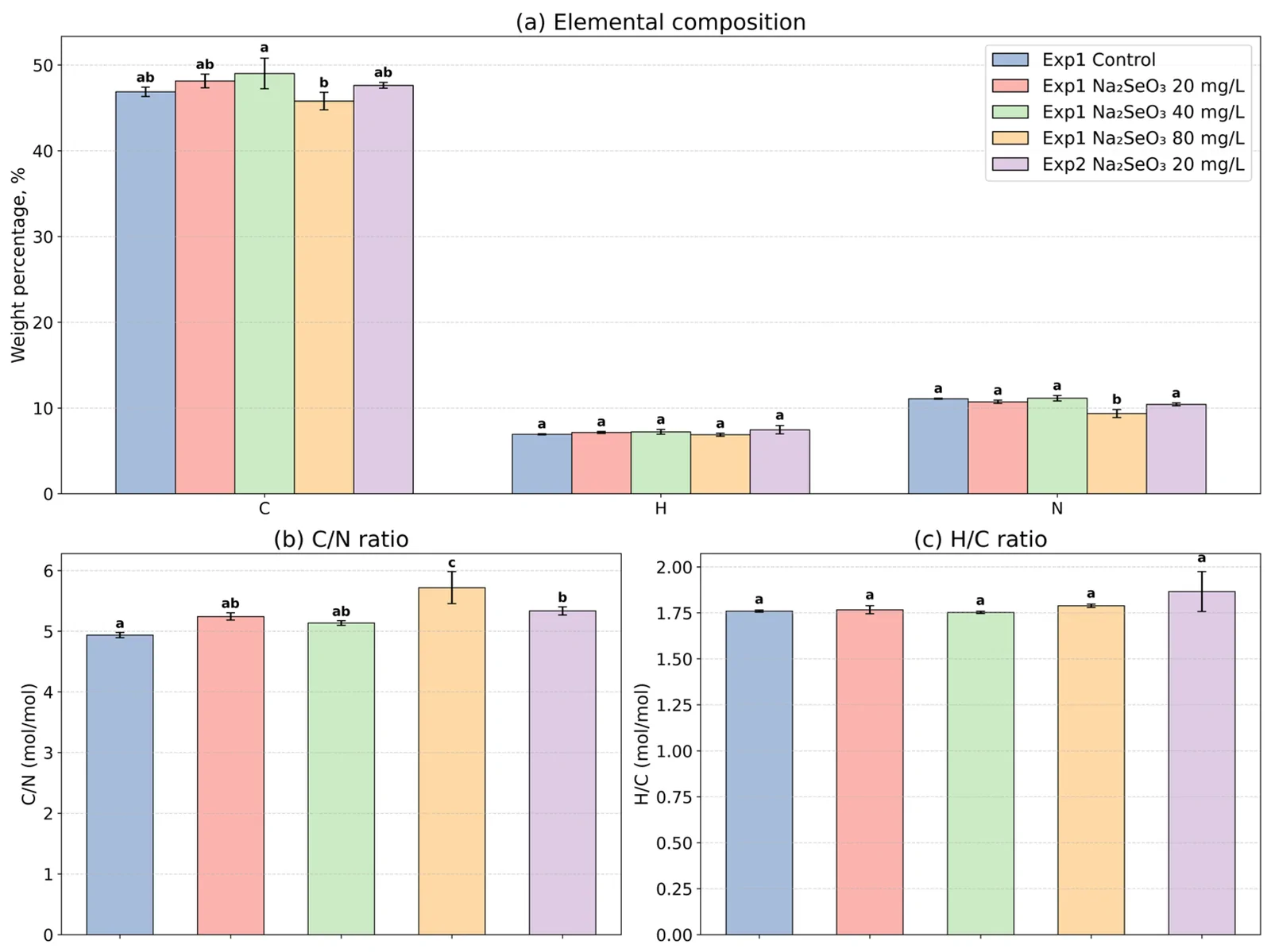
CHNS content (**a**), C/N ratio (**b**), and H/C ratio (**c**) under varying sodium selenite concentrations (mean ± standard deviation, *n* = 3). Columns represent mean values, error bars indicate SD. Statistical analysis: one-way ANOVA followed by Tukey’s HSD post-hoc test (α = 0.05). Different letters above the bars denote statistically significant differences within each panel; groups sharing a common letter are not significantly different.

The results of the CHNS elemental analysis are presented in Figure 10. Statistical analysis (ANOVA) indicated that the sodium selenite concentration in the first experiment exerted a significant effect on all investigated parameters, with the exception of hydrogen content (p=0.139). The most pronounced effects were observed for nitrogen ($\eta^{2}=0.894$) and the C/N ratio ($\eta^{2}=0.867$), which are frequently utilized as indicators of metabolic state. The impact on carbon content and the H/C ratio was also statistically significant (p=0.036 and p=0.035, respectively), although these changes were less pronounced ($\eta^{2}=0.638$ and $\eta^{2}=0.639$, respectively).

Tukey’s post-hoc pairwise comparisons demonstrated that significant deviations from the control occurred exclusively at the maximum sodium selenite concentration (80 mg/L): at this threshold, the nitrogen content was significantly lower, and the C/N ratio was significantly higher, than in the other groups (p<0.05). No statistically significant shifts were observed at the Se20 and Se40 concentrations.

A comparison between the first and second experiments at a sodium selenite concentration of 20 mg/L revealed no statistically significant differences (t-test, p>0.05 for all metrics). This indicates that adaptation did not alter the elemental composition at this specific concentration.

## 3. Discussion

### 3.1. Biomass Productivity, Selenium Accumulation, and the Role of Elevated CO_2_

In the present study, biomass productivity was highest at 10 mg/L Na_2_SeO_3_ (0.369 g·L^−1^·day^−1^), indicating a hormetic response at low selenium concentrations, while higher doses progressively reduced yield, consistent with literature reports of growth inhibition at elevated selenite concentrations [25]. Selenium accumulation in the biomass followed a clear dose-dependent pattern, reaching 762 mg/kg at 80 mg/L. This value substantially exceeds previously reported accumulations (e.g., 220.2 mg/kg at 70 mg/L [9]), which may be attributed to strain-specific differences and, notably, the elevated CO_2_ concentration (3%) used in our study. Elevated CO_2_ likely stimulates culture metabolism, increasing cellular sulfur demand and consequently enhancing the uptake of its chemical analogue, selenium, through shared sulfate transporters [40]. Similar patterns of low-dose stimulation and high-dose inhibition have been reported in other studies of Se-enriched spirulina, where low selenium doses were found to stimulate metabolism, while high concentrations induced oxidative stress, disrupted photosynthetic activity, and suppressed growth [25].

### 3.2. Biochemical Composition: Protein, Lipid, Carbohydrate, and Pigment Responses

Protein content remained stable (60–65%) at moderate selenite concentrations (10–40 mg/L), but decreased significantly to 46.98% at 80 mg/L in the non-adapted culture. This suppression of protein synthesis at high Se concentrations is consistent with previous reports [41] and indicates a metabolic shift away from nitrogenous compound production under selenium stress. The adapted culture (Experiment 2) maintained significantly higher protein content at 80 mg/L (63.49%) compared to the non-adapted culture, demonstrating the adaptive capacity of *L. platensis*.

Lipid accumulation was stimulated at 40 mg/L in the first experiment but reduced at 80 mg/L in the adapted culture, suggesting that selenium-induced lipid peroxidation may occur at lower thresholds under stress conditions [42]. Carbohydrate content showed an increasing trend at high Se concentrations, consistent with previous observations of carbohydrate involvement in selenium binding and biotransformation [41,43]. Phycocyanin content was not significantly affected by selenite treatment, although literature suggests that phycobiliprotein levels may decline under severe oxidative stress at higher Se concentrations [42]. Phenolic content remained unchanged across all treatments, consistent with previous reports that selenium effects on phenolic compounds do not follow a simple dose-dependent pattern [44].

### 3.3. Elemental Composition and Mineral Profile

CHNS analysis revealed that nitrogen content decreased and the C/N ratio increased significantly only at 80 mg/L, confirming a metabolic shift away from protein synthesis toward carbohydrate/lipid accumulation under severe selenium stress [41]. Sulfur was not detected by CHNS analysis, likely because a significant portion of sulfur in spirulina biomass is incorporated into thermally stable sulfated polysaccharides that resist combustion [45].

Sulfur, potassium, and magnesium contents decreased dose-dependently with increasing selenite concentration. The sulfur decline is attributed to competitive inhibition of sulfate transporters by selenite and substitution of sulfur in organic molecules [40]. Potassium reduction is consistent with selenium stress-induced cellular leakage [42]. Magnesium decline was observed in parallel with sulfur and potassium, while calcium showed no clear trend, consistent with published data [46]. Notably, copper content increased anomalously to 19.5 mg/kg at 20 mg/L in the non-adapted culture but normalized to 3.2 mg/kg in the pre-adapted culture (Experiment 2). This indicates that acute selenite stress triggers rapid copper uptake via cell wall adsorption [47], while adapted cultures develop active efflux mechanisms, stabilizing the elemental profile.

Safety assessment according to EC Regulation (EU) 2023/915 revealed that lead, cadmium, chromium, and cobalt remained within regulatory limits across all treatments. However, at 80 mg/L Na_2_SeO_3_, mercury content reached 0.22 mg/kg, exceeding the EU threshold for dietary supplements (0.10 mg/kg), though remaining below the Codex Alimentarius advisory limit (1.0 mg/kg). This elevation may be attributed to spirulina’s high sorption capacity for metal ions [48] and potential stress-induced membrane permeability changes at high Se doses, or trace mercury impurities in the commercial Na_2_SeO_3_ reagent. Nickel levels remained below the EFSA TDI, consistent with literature data [48].

### 3.4. Adaptive Capacity and Practical Implications

A key finding of this study is the enhanced metabolic stability of the pre-adapted culture (Experiment 2), which maintained protein content at 80 mg/L (63.49% vs. 46.98% in non-adapted culture) and normalized copper accumulation (3.2 mg/kg vs. 19.5 mg/kg). This demonstrates the metabolic plasticity of *L. platensis* and its capacity for selenium adaptation, which can be leveraged to stabilize biomass composition in commercial production.

Based on the integrated assessment of biomass productivity, selenium accumulation, nutritional quality, and elemental safety, Na_2_SeO_3_ concentrations of 10–40 mg/L are recommended for producing Se-enriched biomass. This range ensures compliance with heavy metal safety standards, preserves protein and lipid quality (60–65% and 5.5–7.9%, respectively), and achieves target selenium accumulation levels between 88 and 762 mg/kg depending on the enrichment objectives. Cultivation at 80 mg/L is not recommended due to mercury accumulation exceeding regulatory thresholds, protein suppression, and excessive selenium loading.

Future studies should include a more detailed investigation within the 0–20 mg/L Na_2_SeO_3_ range to identify the optimal concentration for specific application targets, balancing biomass productivity, selenium accumulation, and nutritional quality. Additionally, systematic evaluation of varying CO_2_ concentrations on growth kinetics and exploration of the downstream therapeutic efficacy of the produced biomass should be the subject of further research.

## 4. Materials and Methods

### 4.1. Experimental Setup

The experiments were carried out at the Joint Institute for High Temperatures of the Russian Academy of Sciences (Moscow, Russia) in 2025. The experiment was conducted in a sealed gas chamber with a volume of 12 $m^{3}$, equipped with temperature, humidity, and pressure sensors, as well as gas analyzers ($O_{2}$, CO, ${\mathrm{CO}}_{2}$, ${\mathrm{NH}}_{3}$, ${\mathrm{CH}}_{4}$, ${\mathrm{SO}}_{2}$, and ${\mathrm{NO}}_{2}$). The chamber was connected to ${\mathrm{CO}}_{2}$ cylinders to maintain an elevated carbon dioxide concentration. Initially, the chamber was charged with carbon dioxide to reach an internal concentration of 3 vol.%. This concentration was selected based on the findings of our previous studies [34,36] and to match the pre-adaptation conditions of the spirulina culture, which had been maintained at this specific ${\mathrm{CO}}_{2}$ concentration for more than 6 months. Every two days, the chamber was opened for 30 min to collect suspension samples from the photobioreactors. During sampling, inside the chamber the ${\mathrm{CO}}_{2}$ concentration dropped to atmospheric level (0.04 vol.%). Following sample collection, the chamber was re-sealed, and ${\mathrm{CO}}_{2}$ was supplied from the cylinders until the baseline concentration (3 vol.%) was fully restored. During the two-day intervals between sampling events, no additional ${\mathrm{CO}}_{2}$ was supplied from the cylinders, and the photobioreactors remained enclosed within the sealed volume of the chamber under the elevated ${\mathrm{CO}}_{2}$ atmosphere. A detailed description of the gas chamber configuration is provided in [36].

Within the chamber, cylindrical glass column photobioreactors (PBRs) with a working volume of 10 L each (height: 80 cm) were placed on the floor. The photobioreactors were covered with 20 cm × 20 cm gauze lids. Aerators placed at the bottom of the reactors provided continuous sparging with the gas–air mixture drawn from the chamber atmosphere at a flow rate of 1 L/min. Illumination was supplied continuously (24/7) via LED strips at an intensity of 220 $\mu \mathrm{mol}\cdot m^{-2}\cdot s^{-1}$. The flow rate and light intensity were identical across all PBRs. The temperature inside the reactors was maintained at 27 ± 1 °C.

### 4.2. Experimental Procedure

Two consecutive biomass cultivation experiments were performed, each lasting 8 days.

In the first experiment, the *Limnospira platensis* strain pre-adapted to the elevated ${\mathrm{CO}}_{2}$ concentration (3 vol.%) was used as the inoculum. Sodium selenite (anhydrous ${\mathrm{Na}}_{2}{\mathrm{SeO}}_{3}$, 98.5%, RusHim (Moscow, Russia), TU 6-15-0207523-65-88) was introduced as a single dose at the beginning of the experiment at concentrations of 10, 20, 40, and 80 mg/L. The first experiment involved 20 photobioreactors: 4 control reactors (without selenite) and 4 replicates for each of the designated sodium selenite concentrations (10, 20, 40, and 80 mg/L).

In the second experiment, the biomass obtained from the 20 mg/L sodium selenite group of the first experiment served as the inoculum. This design was chosen to evaluate the adaptive capacity of *L. platensis* to prolonged selenium exposure and to assess whether pre-adaptation could stabilize the biochemical and elemental composition under subsequent selenite stress at concentrations of 20, 40, and 80 mg/L.

Sodium selenite was added at the beginning of the experiment at concentrations of 20, 40, and 80 mg/L; photobioreactors without selenite supplementation were also included. The second experiment involved 12 photobioreactors: 3 control reactors (without selenite) and 3 replicates for each of the designated sodium selenite concentrations (20, 40, and 80 mg/L).

**Figure 11:**
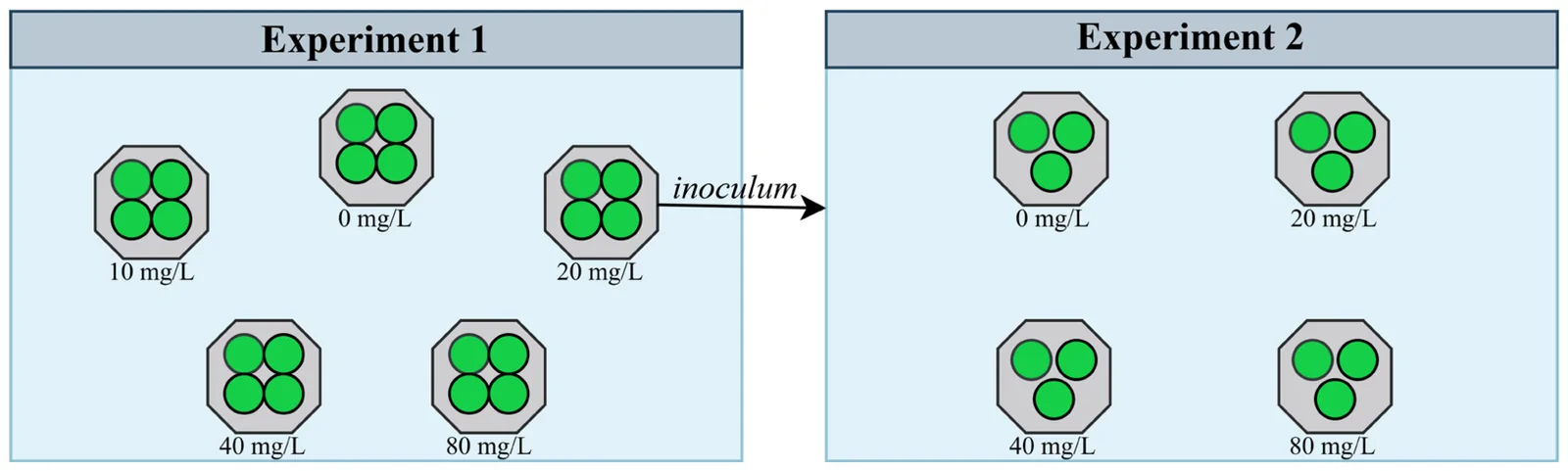
Experimental design indicating the number of photobioreactors and sodium selenite concentrations. In Experiment 2, the biomass cultivated at 20 mg/L Na_2_SeO_3_ in Experiment 1 served as the inoculum.

The experimental design, including the number of photobioreactors and sodium selenite concentrations, is shown in Figure 11.

#### 4.2.1. Strain Adaptation and Cultivation

The study utilized a *Limnospira platensis* strain adapted to an elevated ${\mathrm{CO}}_{2}$ concentration. Adaptation was performed via continuous cultivation for 6 months in an atmosphere containing 3 vol.% ${\mathrm{CO}}_{2}$ [37]. A description of the initial (non-adapted) *Limnospira platensis* strain is provided in our previous works [38,49].

Cultivation was carried out using a modified Zarrouk medium prepared with distilled water. The composition of the medium was as follows (g/L): NaHCO_3_—12.68; KNO_3_—3.0; K_2_HPO_4_·3H_2_O—0.66; K_2_SO_4_—1.0; MgSO_4_·7H_2_O—0.2; NaCl—1.0; CaCl_2_—0.04; FeSO_4_·7H_2_O—0.018; EDTA—0.08; and trace elements—1 mL/L.

#### 4.2.2. Biomass Filtration and Washing

On day 8, the biomass was harvested by filtration through stainless steel sieves with a mesh size of 100 μm. Owing to the large size of the trichomes (up to 800 μm), the filtration of *L. platensis* proceeds via gravity-driven flow. The filtrate obtained after primary filtration was collected for residual nutrient analysis, whereas the sediment (dense pulp) was subjected to washing for salt removal. For the washing procedure, the pulp was mixed with potable water (conforming to SanPiN 2.1.4.1116-02 domestic standard) at a pulp-to-water mass ratio of 1:2. The resulting suspension was re-filtered through the 100 μm stainless steel sieves. The retentate obtained after secondary filtration was transferred to a drying oven to determine the dry weight and analyze the chemical composition.

To ensure sufficient and homogeneous material for the full set of biochemical analyses, the biomass from all replicate photobioreactors within each treatment was pooled, filtered through one sieve, and dried in one tray.

#### 4.2.3. Drying

The filtered biomass was frozen at –20 °C for 4 h in the chamber of a lyophilizer. Subsequently, the frozen samples were freeze-dried at 35 °C until a constant weight was achieved.

### 4.3. Analytical Methods

#### 4.3.1. Determination of Biomass Yield

The final dry biomass concentration of spirulina was calculated using empirical Equation (1), where the optical density (OD) was multiplied by a coefficient of $0.85\;g\cdot L^{-1}$ per OD unit:

(1) $$\begin{array}{cccc} & C_{\mathrm{spirulina}}=OD\times 0.85 & & \end{array}$$

The specific growth rate (μ, ${\mathrm{day}}^{-1}$) was determined for consecutive time intervals (0–2, 2–4, 4–6, and 6–8 days) based on the changes in biomass concentration according to Equation (2):

(2) $$\begin{array}{cccc} & \mu =\frac{\ln\left(\frac{C_{\mathrm{spirulina}2}}{C_{\mathrm{spirulina}1}}\right)}{\Delta t} & & \end{array}$$

Additionally, the average daily biomass productivity ($\mathrm{mg}\cdot L^{-1}\cdot {\mathrm{day}}^{-1}$) was calculated for each interval as the ratio of $\Delta C_{\mathrm{spirulina}}$ to Δt.

#### 4.3.2. Determination of Protein Content

Total protein content was determined using the Lowry method [50]. The method is based on the formation of a copper ion–protein complex in an alkaline medium (biuret reaction) and the subsequent reduction of the Folin–Ciocalteu reagent by the aromatic amino acid residues of the protein. The color intensity was measured spectrophotometrically and calculated using a standard calibration curve. All samples were analyzed in triplicate.

#### 4.3.3. Determination of Lipid Content

Lipids were extracted from the dry biomass using a chloroform:methanol (2:1) mixture according to a modified Bligh and Dyer method [51,52]. Following extraction, phase separation was achieved by centrifugation using an MPW-351R centrifuge (MPW Med. Instruments, Warsaw, Poland). The lipid-containing organic phase was separated and evaporated to a constant weight at 70 °C in a Binder ED 115 drying oven (Binder GmbH, Tuttlingen, Germany). Total lipid content was determined gravimetrically and calculated using Equation (3):

(3) $$Lipids(\%)=\frac{m_{2}-m_{1}}{m_{0}}\times 100$$

where $m_{0}$ is the initial mass of the dry biomass sample, $m_{1}$ is the mass of the empty vessel, and $m_{2}$ is the mass of the vessel containing the extracted lipids.

#### 4.3.4. Determination of Carbohydrate Content

Following the removal of the lipid fraction, the remaining biomass residue was used to determine the total carbohydrate content via the phenol–sulfuric acid method described by Dubois [53]. This method is based on the dehydration of carbohydrates by concentrated sulfuric acid to form furfural derivatives, which subsequently react with phenol to produce colored complexes. The optical density was measured spectrophotometrically, and the carbohydrate content was calculated using a standard calibration curve constructed from glucose standard solutions. The results were expressed as a percentage of the dry biomass weight. All samples were analyzed in triplicate.

#### 4.3.5. Determination of Phycocyanin Content

Phycocyanin extraction was conducted as follows: 1 g of spirulina was suspended in 20 mL of distilled water and placed in an ultrasonic bath at 30 °C for 1.5 h. The samples were then centrifuged at 5000 rpm at 4 °C for 15 min. Absorbance was measured using a UV-1200 spectrophotometer (Mapada, Shanghai, China) at wavelengths of 620 nm, 652 nm, and 280 nm. All samples were analyzed in quadruplicate.

The phycocyanin (C-PC) concentration in the diluted sample ($\mathrm{mg}\cdot {\mathrm{mL}}^{-1}$) was calculated using Equation (4) [54]:

(4) $$C_{PC}=\frac{A_{620}-0.474\times A_{652}}{5.34}$$

where ${\mathrm{OD}}_{620}$ is the optical density at a wavelength of 620 nm and ${\mathrm{OD}}_{652}$ is the optical density at a wavelength of 652 nm.

The extract purity (the ratio of phycocyanin to the total concentration of aromatic proteins) was calculated using Equation (5):

(5) $$Purity=\frac{A_{620}}{A_{280}}$$

where ${\mathrm{OD}}_{280}$ is the optical density at a wavelength of 280 nm.

#### 4.3.6. Determination of Total Phenolic Content

The total phenolic content in the cyanobacterial biomass was determined by the Folin–Ciocalteu method [55]. Phenolic compounds were extracted from the dry biomass using an 80% ethanol solution, followed by ultrasonication and centrifugation. When necessary, acetone precipitation was applied to remove protein interferences.

For the assay, the Folin–Ciocalteu reagent and a 20% sodium carbonate solution were added to the diluted extract. The mixture was incubated in the dark at room temperature for 2 h, after which the optical density was measured spectrophotometrically at 760 nm. Quantitative determination was performed using a calibration curve constructed with gallic acid as the standard. The results were expressed as mg of gallic acid equivalents (GAE) per gram of dry biomass. All samples were analyzed in triplicate.

#### 4.3.7. Trace Element Composition Analysis

Elemental concentrations were determined using an inductively coupled plasma mass spectrometer (ICP-MS, X-7, Thermo Scientific, Waltham, MA, USA). Prior to mass spectral analysis, the samples were digested in acids (using either an open system or an autoclave). The resulting solution, with an analyte concentration of 0.05–0.2%, was nebulized with argon and introduced into the plasma as an aerosol. Within 2 ms, desolvation, vaporization, atomization, excitation, and ionization of the particles occurred in the plasma. Ions from the central zone of the plasma were directed through the interface into the vacuum stage of the mass spectrometer, where a positive ion beam was formed, and photons and neutral particles were deflected. Subsequently, the ions were separated in a quadrupole analyzer based on their mass-to-charge ratio. The ion flux intensity was registered, and the mass spectra were stored on a computer. The samples were analyzed in duplicate.

#### 4.3.8. CHNS Analysis

The determination of nitrogen, carbon, hydrogen, and sulfur content in the cyanobacteria was performed using a Flash 2000 multi-element analyzer (Thermo Scientific, Milan, Italy). The carbon concentration was determined from the chromatograms of the products of catalytic combustion of the sample at a temperature of 900 °C with oxygen delivery to the combustion chamber. The combustion time was 6 s, and the chromatogram recording time was 10 min. The weight of the analyzed sample ranged from 1 to 3 mg (weighing precision of 0.01 mg). Sulfanilamide was used for calibration. The oxygen fraction was calculated by subtracting the sum of the measured carbon, hydrogen, nitrogen, and sulfur concentrations from 100%. To obtain statistically significant results, three parallel measurements were performed.

#### 4.3.9. Statistical Data Processing

Statistical data processing was performed in the Python 3.13.13 environment using the pandas 3.0.3, numpy 2.4.6, scipy 1.17.1, statsmodels 0.14.6, matplotlib 3.10.9, and seaborn 0.13.2 libraries. All data are presented as the arithmetic mean ± standard deviation (M ± SD). The significance level for all statistical tests was set at *p* less than 0.05.

Prior to conducting parametric tests, the normality of the distribution (Shapiro–Wilk test) and the homogeneity of variances (Levene’s test) were verified. If the assumptions were violated, robust methods (Welch’s ANOVA) or non-parametric criteria (Kruskal–Wallis test) were used. One-way analysis of variance (ANOVA) was applied to identify the significance of sodium selenite application effect on the measured variables. Upon detecting statistically significant effects, multiple pairwise comparisons were performed using Tukey’s post-hoc test (in the case of homogeneous variances) or the Games–Howell test with Holm’s correction (if homogeneity was violated). For pairwise comparisons conducted within the framework of the non-parametric Kruskal–Wallis test, a multiple comparison correction was applied using the Benjamini–Hochberg false discovery rate (FDR) method. Student’s *t*-test for independent samples was used to compare two groups. Outliers were identified using the interquartile range (IQR) method with a multiplier of 1.5 and were excluded from further analysis.

## 5. Conclusions

In this study, we investigated the biochemical and elemental profile of *Limnospira platensis* biomass cultivated under elevated CO_2_ (3 vol.%) with sodium selenite supplementation at concentrations of 10, 20, 40, and 80 mg/L, including an assessment of adaptive responses in a pre-cultured inoculum.

It was established that selenium accumulation in the biomass follows a clear dose-dependent pattern: increasing the ${\mathrm{Na}}_{2}{\mathrm{SeO}}_{3}$ concentration in the medium from 10 to 80 mg/L causes the cellular Se content to rise from 88.7 to 762.0 mg/kg.

Moderate selenite concentrations (10–40 mg/L) maintain heavy metal contents within safe regulatory thresholds and do not induce a substantial decline in biomass quality regarding protein and lipid contents.

The most severe disruptions to biochemical and elemental parameters occur at 80 mg/L. This concentration triggers a statistically significant reduction in protein content (down to 46.98% in the first experiment), an accumulation of mercury that exceeds the established safety threshold (0.22 mg/kg vs. the limit of 0.10 mg/kg), an elevation in carbohydrates, and a corresponding shift in the C/N ratio.

A key finding of this study is the distinct behavior observed between the first and second experiments, demonstrating that the pre-adapted culture exhibits higher metabolic stability. In the second experiment, the adapted culture prevented protein degradation at 80 mg/L and normalized the copper content (dropping it from an anomalous 19.5 mg/kg down to 3.2 mg/kg). This underscores the metabolic plasticity of spirulina and its capacity for selenium adaptation, which can be leveraged to stabilize biomass composition in commercial production.

Comparison with external literature confirms that the recorded values for lipids, proteins, carbohydrates, and overall elemental composition generally fall within reported reference ranges for spirulina.

Based on these findings, ${\mathrm{Na}}_{2}{\mathrm{SeO}}_{3}$ concentrations of 10–40 mg/L are recommended for producing selenium-enriched biomass. This range ensures compliance with heavy metal safety standards, preserves protein and lipid quality, and achieves target selenium accumulation levels between 88 and 762 mg/kg depending on the enrichment objectives. Cultivation at 80 mg/L is not recommended due to the risks of regulatory mercury contamination, protein suppression, and excessive selenium loading.

Future studies should include a more detailed investigation within the 0–20 mg/L Na_2_SeO_3_ range to identify the optimal concentration for specific application targets, balancing biomass productivity, selenium accumulation, and nutritional quality.

Further research is required to evaluate the long-term cultivation of spirulina with sodium selenite supplementation and to fully characterize the adaptation kinetics of its biochemical profile during extended cultivation. Overall, this work demonstrates the feasibility of producing selenium-enriched spirulina tailored to specific target concentrations when grown under elevated ${\mathrm{CO}}_{2}$ (3%). Future studies should focus on a more granular investigation within the 0–40 mg/L selenite range, systematically evaluate the specific impacts of varying ${\mathrm{CO}}_{2}$ concentrations on growth kinetics, and explore the downstream therapeutic efficacy of the biomass produced via this developed biotechnology.

## Figures and Tables

**Table 1:** Biomass yield per single PBR ($M_{1\mathrm{PBR}}$) and average daily biomass productivity (V) at various sodium selenite concentrations. Biomass yield per photobioreactor (M_1_PBR) was calculated as the total dry biomass obtained from each treatment group divided by the number of replicate photobioreactors (*n* = 4 for Experiment 1; *n* = 3 for Experiment 2). Average daily productivity (V) was calculated as M_1_PBR divided by the working volume (10 L) and cultivation duration (8 days).

	Experiment 1					Experiment 2			
$C_{{\mathrm{Na}}_{2}{\mathrm{SeO}}_{3}}$ (mg/L)	0	10	20	40	80	0	20	40	80
M_1PBR_ (g)	27.5	29.5	24	20	16.3	21.7	21.3	14.1	7.1
V ($g\cdot L^{-1}\cdot {\mathrm{day}}^{-1}$)	0.312	0.369	0.268	0.218	0.172	0.229	0.224	0.134	0.046
Sample Designation	Se0_1_	Se10_1_	Se20_1_	Se40_1_	Se80_1_	Se0_2_	Se20_2_	Se40_2_	Se80_2_

**Table 2:** Trace element and mineral composition of spirulina samples under varying sodium selenite supplementations. Values below the limit of detection (LOD) are denoted as <LOD.

		Sample Designation/Sodium Selenite Supplementations, mg/L				
		Se0_1_/0	Se10_1_/10	Se20_1_/20	Se20_2_/20	Se80_1_/80
Element	Limit of Detection (μg/g)	Element content, (μg/g)				
Li	0.003	0.061	0.056	0.074	0.062	0.057
B	0.3	1.4	1.5	1.6	1.3	2.1
Na	2	9944	10,997	10,486	10,433	10,934
Mg	0.4	2830	2644	2696	2393	2097
Al	1	12.7	11.4	44.9	26.4	36.7
P	1	9373	8578	9044	8128	7472
S	2	8312	8179	7346	7648	6563
K	1	21,722	21,388	18,393	18,240	14,101
Ca	4	925	869	1066	981	812
Ti	0.1	0.41	<LOD	1.0	0.58	1.1
Cr	0.1	0.73	0.73	2.6	1.6	2.2
Mn	0.02	28.8	30.4	30.7	35.0	35.0
Fe	0.3	385	392	408	363	332
Co	0.02	0.33	0.22	0.31	0.19	0.25
Ni	0.1	1.0	1.2	0.62	1.1	0.99
Cu	0.1	2.9	4.1	3.2	19.5	7.3
Zn	0.1	14.9	16.3	15.6	17.6	14.5
Ga	0.004	0.044	0.039	0.77	0.51	0.68
As	0.01	<LOD	<LOD	<LOD	<LOD	<LOD
Se	0.02	0.50	88.7	439	394	762
Rb	0.01	12.7	12.7	6.7	7.2	6.9
Sr	0.01	8.6	7.6	8.2	6.6	6.5
Mo	0.005	0.27	0.26	0.60	0.43	0.50
Cd	0.002	0.003	<LOD	0.004	0.004	0.005
Sn	0.01	0.053	0.059	0.051	0.13	0.068
Sb	0.01	<LOD	<LOD	0.019	<LOD	0.087
Cs	0.001	0.0038	0.0040	0.0034	0.0028	0.0033
Ba	0.01	0.63	0.57	1.2	0.78	1.0
La	0.002	0.017	0.0058	0.014	0.008	0.011
Ce	0.003	0.080	0.090	0.14	0.11	0.20
Hg	0.003	0.005	0.027	0.058	0.048	0.22
Tl	0.001	0.0020	0.0017	0.0023	0.0025	0.0026
Pb	0.006	0.042	0.037	0.053	0.098	0.053

## Data Availability

The original contributions presented in the study are included in the article material, further inquiries can be directed to the corresponding author.
